# [Corrigendum] Downregulation of Notch1 induces apoptosis and inhibits cell proliferation and metastasis in laryngeal squamous cell carcinoma

**DOI:** 10.3892/or.2023.8581

**Published:** 2023-06-06

**Authors:** Meng-Yuan Dai, Fang Fang, You Zou, Xing Yi, Yong-Jun Ding, Chen Chen, Ze-Zhang Tao, Shi-Ming Chen

Oncol Rep 34: 3111–3119, 2015; DOI: 10.3892/or.2015.4274

Following the publication of this article, a concerned reader drew to the authors' attention that a pair of the 24 h scratch-wound assay data panels in Fig. 4A, and three of the migration and invasion assay data panels in Fig. 4B, exhibited overlapping sections, suggesting that data which were intended to have shown the results from differently performed experiments had originated from the same sources. In addition, the total number of cases for the LSCC sample data in Table II did not reflect the sum of the samples indicated in the ‘negative’, ‘positive’ and ‘strong positive’ categories.

After having consulted their original data, the authors have realized that Table II and Fig. 4 contained some inadvertent errors: The authors divided their control group data into two subgroups, namely the non-transfection and negative-shRNA groups, although they overlooked details of the filing system they had devised for saving the data, and mistakenly included images from the non-transfection group in with the negative-shRNA group due to unclear file labeling. Moreover, in Table II, the data value for the ‘positive’ stained samples should have been written as ‘43’, not ‘44’.

The corrected versions of Table II and Fig. 4, which now shows the corrected data for the ‘Negative-shRNA / 24 h’ experiment in Fig. 4A and the ‘Non-transfection / Invasion’ and ‘Negative-shRNA / Migration’ experiments in Fig. 4B, are shown below and on the next page, respectively. The authors sincerely apologize for the errors that were introduced during the preparation of this table and this figure, thank the Editor of *Oncology Reports* for granting them the opportunity to publish this corrigendum, and regret any inconvenience that these mistakes may have caused to the readership.

## Figures and Tables

**Figure 4.**
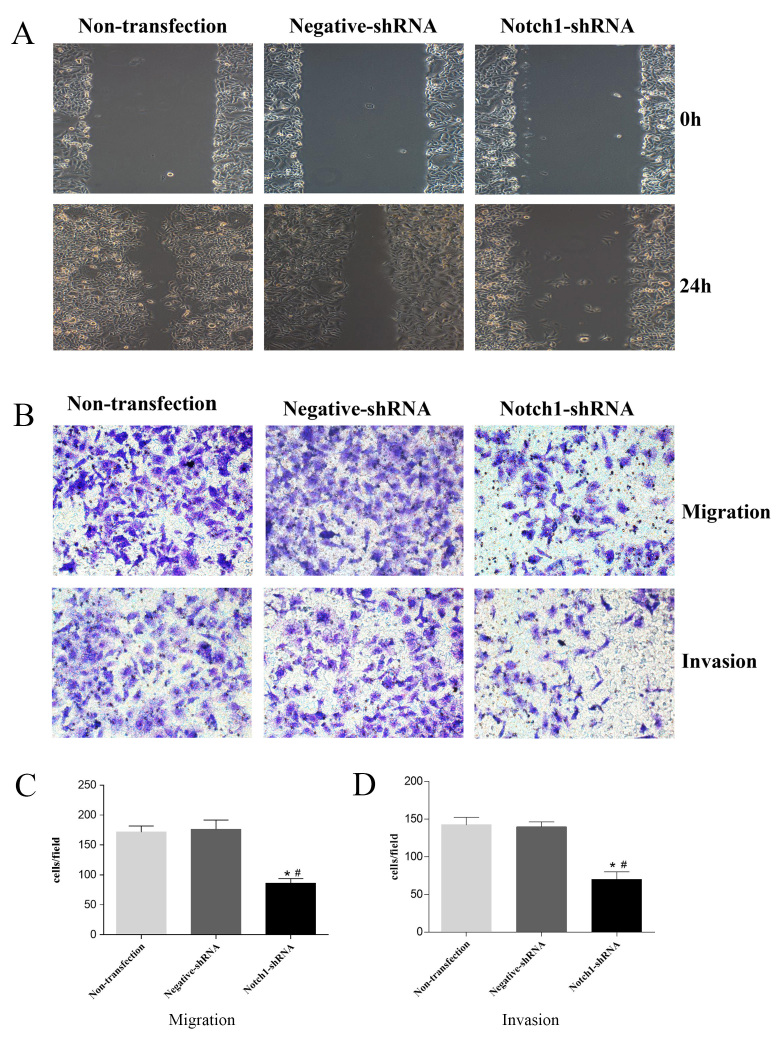
Notch1 knockdown inhibits HEp-2 cell migration and invasion abilities. (A) Photomicrograph of scratches made in the shRNA-transfected HEp-2 cell layer showing inhibition of cellular motility in the Notch1-silenced cells as compared with the non-transfected and negative shRNA groups. (B) Representative images of the Transwell assay without (top panel) or with (bottom panel) Matrigel coating after shRNA transfection. (C) Migration assay results showing the number of HEp-2 cells that had migrated through the non-Matrigel coated filters. (D) invasion assay showing the number of cells that had passed through the Matrigel-precoated filters. The cell counts presented are the mean values/field from at least five randomly selected low-power fields (magnification, ×200) from three independent experiments (error bars, means ± SD). *P<0.05 compared with the non-transfected group; ^#^P<0.05 compared with the negative shRNA group.

**Table II.** QD-IHC detection of Notch1 expression in LSCC and normal vocal polyp tissues.

Sample	Case	Negative	Positive	Strong positive	P-value
Normal vocal polyps	31	24	5	2	
LSCC	95	11	**43**	41	<0.01^a^
Without metastasis	72	11	39	22	<0.01^a^
With metastasis	23	0	4	19	<0.01^b^

a When compared with normal vocal polyp tissues.

b When compared with normal vocal polyp tissues and LSCC without metastasis. QD-IHC, quantum dot immunohistochemistry; LSCC, laryngeal squamous cell carcinoma.

